# Replacing true non-contrast imaging with DECT in GI bleeding demonstrates non-inferior diagnostic performance, reading time and confidence

**DOI:** 10.1007/s00330-025-12191-y

**Published:** 2025-12-17

**Authors:** Moritz Oberparleiter, Hanns-Christian Breit, Jan Vosshenrich, Alina C. Seifert, Paul Hehenkamp, Sonaz Malekzadeh, Adrian Kobe, Daniel T. Boll, Christoph J. Zech, Markus M. Obmann

**Affiliations:** 1 Department of Radiology, University Hospital Basel, University of Basel, Basel, Switzerland; 2 Department of Diagnostic and Interventional Radiology, Sion Hospital, Sion, Switzerland

**Keywords:** Gastrointestinal hemorrhage, Intestines, Tomography (X-ray computed), Radiation dosage

## Abstract

**Objectives:**

To determine whether a dual-energy CT (DECT) protocol—including arterial and portal-venous phases, virtual non-contrast (VNC) images, and iodine maps—provides non-inferior diagnostic performance to a conventional triphasic CT protocol for gastrointestinal (GI) bleeding.

**Materials and methods:**

In this retrospective single-center diagnostic accuracy study, we included all patients who underwent triphasic abdominal CT for GI bleeding between September 2015 and June 2024. For each case, conventional and DECT datasets were generated. Three fellowship-trained abdominal radiologists and two residents independently assessed all cases for active GI bleeding. A consensus review served as the reference standard. Sensitivity and specificity were compared using the Wald method for paired confidence intervals (non-inferiority margin 3%). Diagnostic confidence and reading time were analyzed using the Wilcoxon signed-rank test. Inter-reader agreement was assessed with Fleiss’ kappa.

**Results:**

One hundred patients (mean age, 70 ± 14 years; 34 women) were evaluated, including 50 with GI bleeding (21 upper, 29 lower) and 50 controls. With conventional triphasic CT, sensitivity and specificity were 91.6% and 94.4%, respectively. With DECT, they were 94.4% and 96.0%, demonstrating non-inferiority within a 3% margin. Diagnostic confidence increased from 4 (IQR, 4–5) to 5 (IQR, 4–5) (*p* < 0.01). Mean reading time decreased from 96.3 s to 93.6 s (*p* < 0.01), also meeting non-inferiority. Inter-reader agreement was almost perfect (κ = 0.82). Total DLP was reduced by 20% when true non-contrast images were omitted.

**Conclusion:**

DECT-derived VNC and iodine maps provide non-inferior diagnostic performance to conventional CT for GI bleeding and can replace true non-contrast imaging.

**Key Points:**

***Question*** *Eliminating true non-contrast scans from CT protocols for gastrointestinal bleeding could reduce radiation dose, but its impact on diagnostic performance remains unclear.*

***Findings*** *Dual-energy CT with virtual non-contrast images and iodine maps achieved non-inferior sensitivity, specificity, reading time, and diagnostic confidence compared to conventional triphasic CT.*

***Clinical relevance*** *Dual-energy CT with virtual non-contrast images and iodine maps can reliably replace true non-contrast scans in GI bleeding protocols, maintaining diagnostic performance while reducing radiation exposure—offering a safer and more efficient diagnostic alternative for patients.*

**Graphical Abstract:**

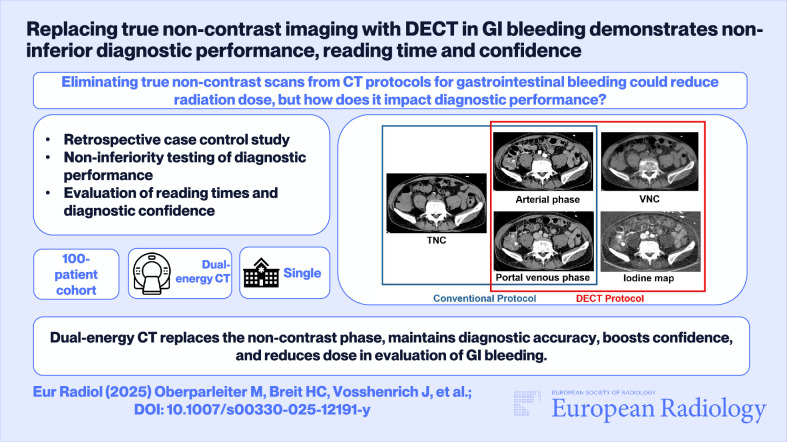

## Introduction

Imaging, especially triphasic CT angiography (CTA), is an important diagnostic tool in both upper and lower gastrointestinal (GI) bleeding. CTA often complements endoscopy in cases of upper GI bleeding, particularly if endoscopy cannot establish the origin of bleeding [1]. Further, when endoscopy is contraindicated due to recent surgery or after trauma, CTA or conventional angiography are the modalities of choice [1]. In cases of lower GI bleeding in hemodynamically stable patients, colonoscopy, CTA, and technetium-99m–labeled red blood cell scans are usually considered appropriate for diagnostics [2]. However, in hemodynamically unstable patients and patients requiring numerous blood transfusions, CTA is recommended, while the role of colonoscopy is debated [2].

Several reviews have discussed the role of dual-energy CT (DECT) in GI bleeding [3–5]. It has been hypothesized that using DECT-derived virtual non-contrast (VNC) images and iodine maps improves diagnostic performance and reader confidence, while radiation dose and scan time can be reduced when true non-contrast scans are omitted [3]. However, only one dated clinical study with a medium-sized patient cohort exists that evaluated DECT in GI bleeding [6, 7]. Sun et al found no statistically significant superiority of the DECT protocol over the triphasic protocol and did not test for non‑inferiority [6, 7]. To our knowledge, no studies have compared the diagnostic confidence and reading times of the DECT protocol to those of the triphasic protocol.

Despite the limited evidence, current guidelines recommend replacing true non-contrast images with VNC images in suspected upper GI bleeding [2]. In contrast, guidelines for suspected lower GI bleeding do not mention DECT [3].

We aimed to evaluate whether a DECT protocol—comprising arterial and portal-venous phases, VNC images, and iodine maps—offers non-inferior diagnostic performance for GI bleeding compared to a conventional triphasic protocol. For this purpose, we conducted a retrospective multireader, case-control study including 100 patients: 50 with active GI bleeding and 50 matched controls. Additionally, we assessed whether reading time and diagnostic confidence were non-inferior. Given the improved conspicuity of contrast extravasation in iodine maps, we further explored whether the DECT protocol might offer superior diagnostic performance, particularly in terms of sensitivity. Finally, we estimated the potential for radiation dose reduction by omitting true non-contrast scans.

## Materials and methods

This retrospective, single-center study was approved by the institutional review board (IRB), and the need for informed consent was waived.

### Study population

**Fig. 1:**
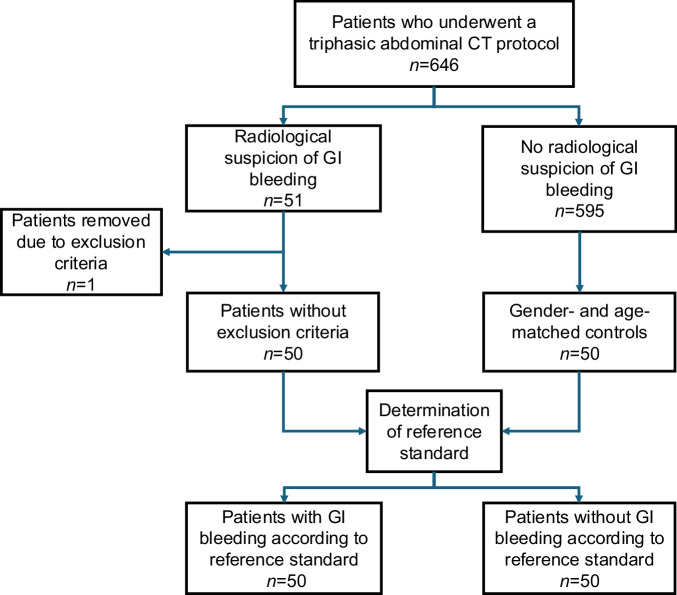
Flowchart of the recruitment of the study population

We searched our picture archiving and communication system (PACS) for all patients who underwent triphasic (true non-contrast, arterial, and portal-venous phase) abdominal dual-energy CT protocol for suspected bleeding at the University Hospital Basel, a tertiary referral center in Switzerland, between September 2015 and June 2024 (Fig. 1). This represents the standard CT protocol for suspected gastrointestinal hemorrhage at our institution. Initially, 646 consecutive patients were identified. We searched the reports of all scans for the radiological diagnosis of acute GI bleeding (*n* = 51). Scans of 595 patients had no radiological suspicion of active GI bleeding.

We excluded patients meeting any of the following criteria from the study: (1) patients younger than 18 years, (2) documented refusal of the patient, (3) oral contrast agent administration, or (4) incomplete imaging data.

The remaining 50 patients were gender- and age-matched with 50 controls. We recruited the controls from the group of patients without radiological suspicion of GI bleeding, taking the exclusion criteria into account. Patients with GI bleeding were matched to those without suspicion of bleeding with the smallest age difference at the time of CT imaging. Gender was the secondary matching criterion. For all patients and controls, laboratory data, including hemoglobin, hematocrit, platelet count, INR and CRP, were extracted from the medical records.

### CT image acquisition

Abdominal DECT was performed on two dual-source, 192-slice scanners (SOMATOM Force, Siemens Healthineers). As part of our standardized imaging protocol for GI bleeding (Table 1), each patient underwent a non-contrast (100 kVp), arterial (70–100 kVp), and portal-venous phase; the latter used dual-energy 100/150 kVp with tin filtration, with tube current modulated by automatic exposure control. Intravenous contrast (Iopamidol 370, Bracco or Iopromide 370, Bayer) was administered at 1.1–1.3 mL/kg body weight (70–115 mL total) with a flow rate of 3–4 mL/s. Arterial scanning began 9 s after a 100 HU aortic trigger; portal-venous scanning followed 55 s later.

**Table 1:** Characteristics of the 50 patients with GI bleeding and the 50 control subjects

	Overall (*n* = 100)	Bleeding (*n* = 50)	Controls (*n* = 50)	*p*-values
Age	70.3 ± 14.3	70.2 ± 14.1	70.4 ± 14.7	0.49
Female/male	34 (34) / 66 (66)	15 (30) / 35 (70)	19 (38) / 31 (62)	0.22
Body mass index (kg/m^2^)	25.5 ± 4.3	25.5 ± 4.6	25.5 ± 4.1	0.96
Emergency admissions/Inpatients	44 (44) / 56 (56)	21 (42) / 29 (58)	23 (46) / 27 (54)	0.82
Laboratory data				
Hemoglobin (g/dL)	8.8 ± 2.0	8.7 ± 1.7	9.0 ± 2.3	0.49
Hematocrit (%)	27 ± 8	26 ± 6	29 ± 11	0.28
Platelet count (10⁹/L)	228 ± 150	232 ± 136	224 ± 164	0.95
INR	1.4 ± 0.5	1.4 ± 0.5	1.3 ± 0.4	0.18
CRP (mg/mL)	62 ± 71	58 ± 60	65 ± 80	0.53
Location of bleeding				
Upper GI bleeding	-	21 (42)	-	-
Lower GI bleeding	-	29 (48)	-	-

Numbers in parentheses represent percentages. Values are expressed as means ± standard deviation

*INR* international normalized ratio, *CRP* C-reactive protein

Images were reconstructed as follows: arterial phase, 1.5 mm slices/1 mm increment (medium soft-tissue kernel, Br36); non-contrast and portal-venous phases, 120 kVp-equivalent mixes at 5 mm slices/2.5 mm increment (Br36). VNC images and iodine maps were generated automatically using a dedicated dual-energy post-processing software (Syngo.via, version VB40A, Siemens Healthineers) from the 100/150 kVp data (5 mm slices /2.5 mm increment, Br36). All datasets used a 512 × 512 matrix, with sagittal and coronal reformats provided for every phase except VNC and iodine maps.

### CT Image interpretation

**Fig. 2:**
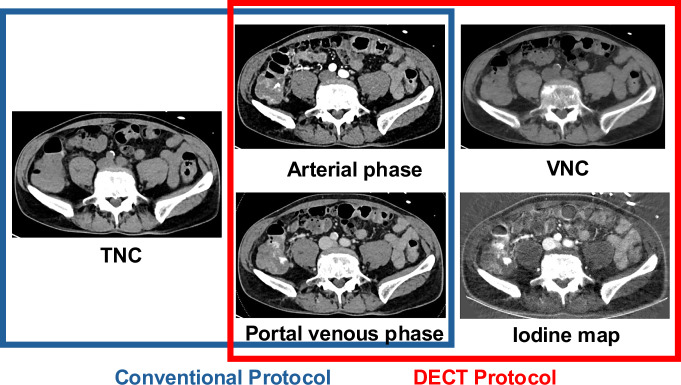
Composition of the conventional and DECT protocol. The conventional protocol comprises a true non‑contrast (TNC) phase, arterial and portal‑venous phases. In the DECT protocol, the TNC acquisition is omitted and replaced by virtual non‑contrast (VNC) images generated from the dual‑energy dataset; iodine maps are supplied as supplementary information, while the arterial and portal‑venous phases remain unchanged

Five readers independently assessed images from both the conventional and DECT protocols (Fig. 2). We blinded all readers to clinical information, prior radiological reports and the reference standard. Three readers were fellowship-trained abdominal radiologists with 6, 8 and 9 years of experience. Two readers were residents in their second and third year of training. The reading was performed using the clinical PACS (IDS7, Sectra).

The scans were anonymized and presented to the readers. The reading was performed in two sessions. In the first session, each reader evaluated images from 50 patients using the DECT protocol and from 50 different patients using the conventional protocol. After a washout period, the second session was conducted, during which each reader assessed the same 100 patients again, but with the alternative protocol (i.e., patients initially reviewed with the DECT protocol were now reviewed with the conventional protocol, and vice versa). In each session, 50 patients with and without GI bleeding were included. This crossover was performed to level any bias due to learning effects. Within both sessions, the cases were presented in random order. A washout period of at least 1 week was maintained between both sets.

The readers were asked to report whether active bleeding was present. For both protocols, active GI bleeding was pre-specified as any contrast extravasation in the arterial phase with venous pooling or venous phase extravasation, not present on the true or virtual non-contrast phase [8]. The assessments were constrained to a binary outcome (bleeding present or absent); indeterminate ratings were not permitted. The readers measured their reading times. Diagnostic confidence was reported using a 5-point Likert scale, where 1 represented the lowest, and 5 represented the highest confidence.

### Reference standard

The reference standard was determined through a consensus review conducted by a fellowship-trained abdominal radiologist and a fellowship-trained interventional radiologist. In cases of disagreement, a senior radiologist with dual fellowship training in abdominal and interventional radiology and 25 years of experience acted as a tiebreaker based on imaging findings and clinical data, including endoscopic evaluation and follow-up examinations. The reference standard assessors were blinded to the assessments of the readers.

### Statistical analysis

Continuous data are presented as means with standard deviation, and discrete data as medians with interquartile ranges. Inter-reader agreement for detecting GI bleeding was determined using Fleiss’ kappa [9]. Sensitivity, specificity, and accuracy were reported as percentages with 95% confidence intervals (CI).

Demographic data, body mass index and laboratory parameters were compared with either Student’s *t*-test or McNemar’s test. Units of transfused packed erythrocyte concentrate, administered over the entire duration of the bleeding management, and necessary therapeutic procedures were recorded as surrogates of bleeding severity.

Non-inferiority of the DECT-derived imaging protocol for sensitivity and specificity was concluded if the lower bound of the two-sided 95% Wald confidence interval for the paired difference exceeded the predefined non-inferiority margin of 3%. The 3% margin was selected based on the effect size observed in a prior study [7]. Further, if non-inferiority was established, we performed superiority testing using McNemar’s test. For diagnostic accuracy, a one-sided Z-test was used to determine non-inferiority, while McNemar’s test was used for superiority testing. No exploratory post hoc analyses of diagnostic performance were conducted; all analyses were pre-specified.

Reading times were reported in seconds, and non-inferiority was tested with a one-sided Wilcoxon signed-rank test, as we consider an increase in reading time of 3 s not clinically significant. Further, if non-inferiority was established, we performed pre-specified superiority testing using the two-sided Wilcoxon signed-rank test for the reading times. In such a case, no corrections for multiple testing are necessary [10]. As defining a non-inferiority margin on the 5-point Likert scale is difficult, we only performed superiority testing for reader confidence with a two-sided Wilcoxon signed-rank test.

For the diagnostic performance metrics, no subgroup analyses were performed, as the anticipated number of discordant cases was considered insufficient for reliable estimation using the Wald method. Nevertheless, performance metrics for the subgroups (senior vs junior readers and upper vs lower GI bleeding) are reported in the “Results” section to inform future research. In contrast, subgroup analyses for reading time and diagnostic confidence (senior vs junior readers and upper vs lower GI bleeding) were conducted, as the corresponding statistical tests are more robust given the sample size. Bonferroni correction was applied to adjust for multiple comparisons in these subgroup analyses. No exploratory post hoc analyses were performed.

*p*-values < 0.05 were considered statistically significant. Statistical analyses were conducted using SPSS Statistics (version 29, IBM) and Python (version 3.9), including the libraries numpy (version 1.20), scipy (version 1.7), and pandas (version 1.3). No formal sample-size calculation was performed; the study included all consecutive patients who met the eligibility criteria. We aimed to include around 100 patients in the final study cohort. Patients with incomplete imaging data were excluded; consequently, no imaging data were missing for either imaging protocol or for the reference-standard assessment.

## Results

The study included 100 patients: 50 with acute GI bleeding and 50 age‑ and gender‑matched controls (Fig. 1). One of 51 initially identified GI bleeding cases was excluded for incomplete imaging. Mean age was 70.3 ± 14.3 years; 34 were women and 66 men, with no significant differences in age, gender, body mass index, and laboratory parameters between both groups (Table 1). No CT-related adverse events were recorded.

Of the 50 patients with active GI bleeding, 19 underwent endoscopic therapy, 16 were managed conservatively, eight required endovascular coiling, six underwent surgery, and one received both endovascular embolization and endoscopic therapy. The mean transfusion requirement amounted to 3 units of packed erythrocyte concentrates (IQR, 2–4).

The reference standard was CT in nearly all cases; endoscopy, performed a mean 4 h after CT (range 3–6 h) without intervening treatment, resolved three equivocal readings.

Controls most often presented with prior but inactive GI bleeding (*n* = 26). Other diagnoses were bleeding of non-gastrointestinal origin (3), colorectal cancer (3), anemia (2), sepsis (2), and single instances of pancreatitis, pneumonia, pyelonephritis, ischemic or ulcerative colitis, small‑bowel obstruction, gastric cancer, acute bowel ischemia, and multiorgan failure; five controls had no identifiable cause.

### Inter-reader agreement

Inter‑reader agreement for active GI bleeding was almost perfect overall (κ = 0.82, 95% CI: 0.78–0.87). With the DECT protocol, the inter-reader agreement was also almost perfect (κ = 0.85, 95% CI: 0.79–0.91), but it was only substantial with the conventional protocol (κ = 0.80, 95% CI: 0.73–0.86). Agreement was κ = 0.87 (95% CI: 0.80–0.94) for upper and κ = 0.79 (95% CI: 0.73–0.84) for lower GI bleeding. Fellowship‑trained radiologists showed κ = 0.87 (95% CI: 0.79–0.95), whereas residents achieved κ = 0.78 (95% CI: 0.64–0.92).

### Diagnostic performance

Overall sensitivity and specificity for diagnosing acute GI bleeding across all readers were 91.6% (95% CI: 87.5–94.7%) and 94.4% (95% CI: 90.8–96.9%), respectively, using the conventional protocol (Table 2). With the DECT protocol, sensitivity was 94.4% (95% CI: 90.8–96.9%) and specificity was 96.0% (95% CI: 92.8–98.1%). The paired difference in sensitivity between DECT and conventional imaging was 2.9%, with a 95% Wald confidence interval of −0.6% to +6.4%. For specificity, the paired difference was 1.6%, with a 95% Wald confidence interval of −1.8% to +5.0%. Using the predefined non-inferiority margin of 3%, non-inferiority was demonstrated for both sensitivity and specificity. However, superiority could not be concluded for sensitivity (*p* = 0.16) and specificity (*p* = 0.48). Overall diagnostic accuracy was 93.0% for the conventional and 95.2% for the DECT protocol. While the difference did not reach statistical significance for superiority (*p* = 0.14), non-inferiority of the DECT dataset was confirmed with a 3% margin (*p* < 0.01).

**Table 2:** Diagnostic performance of conventional vs dual-energy CT protocol for the assessment of GI bleeding

	Conventional	DECT-derived	Difference	*p*-values
	Value (95% CI)	Value (95% CI)	(95% Wald CI)	(Superiority)
Sensitivity	91.6 (87.5–94.7)	94.4 (90.8–96.9)	2.8 (−0.6 to 6.4)	0.16
Specificity	94.4 (90.8–96.9)	96.0 (92.8–98.1)	1.6 (−1.8 to 5)	0.48
Accuracy	93.0 (90.4–95.1)	95.2 (92.9–96.9)	–^a^	0.14

Sensitivity and specificity of the DECT protocol were non-inferior using the 3% margin, as demonstrated by the 95% Wald confidence intervals

All values are presented as percentages

^a^ For accuracy, no 95% Wald CI was calculated, instead non-inferiority was shown with a one-sided Z-test (*p* < 0.01)

Among fellowship-trained abdominal radiologists, sensitivity increased from 94.0% (95% CI: 88.9–97.2%) to 95.3% (95% CI: 90.6–98.1%). In the resident subgroup, sensitivity rose from 88.0% (95% CI: 80.0–93.6%) to 93.0% (95% CI: 86.1–97.1%). Specificity for fellowship-trained radiologists increased from 96.7% (95% CI: 92.4–98.9%) to 97.3% (95% CI: 93.3–99.3%) when using the DECT dataset. Among residents, specificity increased from 91.0% (95% CI: 83.6–95.8%) to 94.0% (95% CI: 87.4–97.8%) (Table 3).

**Table 3:** Diagnostic performance of conventional vs DECT protocol for the detection of GI bleeding

	Conventional	DECT-derived
	Value (95% CI)	Value (95% CI)
Upper GI bleeding		
Sensitivity	93.3 (86.8–97.3)	96.2 (90.5–99.0)
Specificity	95.2 (89.2–98.4)	99.0 (94.8–100)
Accuracy	94.3 (90.2–97.0)	97.6 (94.5–99.2)
Lower GI bleeding		
Sensitivity	90.3 (84.3–96.6)	93.1 (87.7–96.6)
Specificity	93.8 (88.5–97.1)	93.8 (88.5–97.1)
Accuracy	92.1 (88.3–94.9)	93.5 (90.0–96.0)
Senior radiologists		
Sensitivity	94.0 (88.9–97.2)	95.3 (90.6–98.1)
Specificity	96.7 (92.4–98.9)	97.3 (93.3–99.3)
Accuracy	95.3 (92.3–97.4)	96.3 (93.5–98.2)
Residents		
Sensitivity	88.0 (80.0–93.6)	93.0 (86.1–97.1)
Specificity	91.0 (83.6–95.8)	94.0 (87.4–97.8)
Accuracy	89.5 (84.4–93.4)	93.5 (89.1–96.5)

All values are presented as percentages

### Diagnostic confidence

**Fig. 3:**
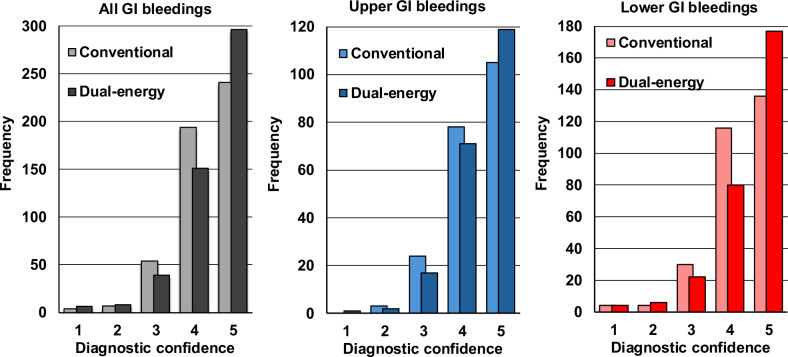
Histogram plots of the distribution of readers’ diagnostic confidence of cases with conventional CT vs DECT protocol. Diagnostic confidence is graded on a 5-point Likert scale

Diagnostic confidence on the 5-point Likert scale was higher with DECT (median 5, IQR 4–5) than with conventional CT (median 4, IQR 4–5) (Fig. 3), demonstrating superiority (*p* < 0.01) and thus non‑inferiority. Fellowship‑trained radiologists showed a similar advantage (median 5, IQR 4–5 for both, but distribution shift; *p* < 0.001), whereas the rise among residents (median 4, IQR 3.25–5 to 4, IQR 4–5) was not significant (*p* = 0.11). For upper‑GI bleeding, confidence increased from 4.5 (IQR 4–5) to 5 (IQR 4–5) without reaching significance after correction for multiple testing (*p* = 0.06); in lower‑GI bleeding, it rose from 4 (IQR 4–5) to 5 (IQR 4–5), achieving significance (*p* < 0.01).

### Reading time

**Fig. 4:**
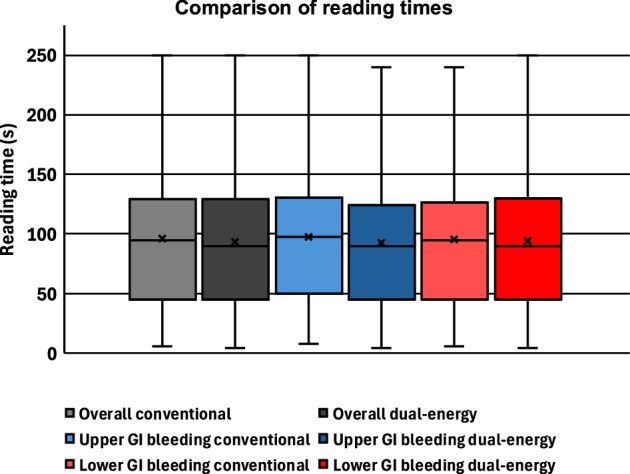
Comparison of reading times of the conventional and DECT protocols

Mean reading time per case fell from 96 ± 54 s with the conventional protocol to 94 ± 58 s with the DECT protocol (Fig. 4). Using the pre-specified 3‑s margin, DECT was non‑inferior (*p* < 0.01) but not superior (*p* = 0.053). Non‑inferiority also held for lower GI bleeding (97 ± 54 vs 93 ± 56 s) and upper GI bleeding (96 ± 55 vs 94 ± 60 s) (both *p* < 0.01); superiority was not reached after correction for multiple testing (*p* = 0.80 and 0.092, respectively). Fellowship‑trained radiologists read marginally slower with the DECT protocol (81 ± 48 vs 80 ± 51 s), yet reading times remained non‑inferior (*p* < 0.01). Residents were faster with the DECT protocol (113 ± 63 vs 120 ± 44 s), demonstrating both non‑inferiority (*p* < 0.01) and superiority (*p* = 0.02).

### Dose reduction

The average DLP was 1431 ± 784 mGy*cm for the total scan and 290 ± 154 mGy*cm for the true non-contrast scans (Table 4). This amounts to a possible dose reduction of 20.4% when non-contrast scans are omitted. The average CTDI_vol_ was 27.08 ± 12.67 mGy for the total scan and 5.69 ± 2.57 mGy for the true non-contrast scans. Similarly, this represents a possible CTDI_vol_ reduction of 21.1%.

**Table 4:** CT acquisition parameters and radiation dose

	Overall (*n* = 100)	Bleeding (*n* = 50)	Controls (*n* = 50)
Contrast			
Contrast volume (mL)	92.8 ± 10.2	92.9 ± 10.0	92.7 ± 10.3
Flow rate (mL/s)	3.92 ± 0.29	3.93 ± 0.22	3.91 ± 0.35
Overall			
CTDI_vol_ (mGy)	27.1 ± 12.7	27.4 ± 13.3	26.8 ± 11.9
DLP (mGy·cm)	1431 ± 784	1490 ± 848	1463 ± 775
True non-contrast			
kVp	100	100	100
Ref. mAs	100	100	100
mAs	141 ± 64	144 ± 67	139 ± 59
CTDI_vol_ (mGy)	5.69 ± 2.57	5.78 ± 2.72	5.60 ± 2.38
DLP (mGy·cm)	290 ± 154	296 ± 164	283 ± 141
Arterial phase			
kVp	88.1 ± 8.7	88.1 ± 8.3	88.2 ± 8.9
Ref. mAs	201 ± 59	201 ± 55	201 ± 61
mAs	277 ± 76	285 ± 87	269 ± 63
CTDI_vol_ (mGy)	8.04 ± 4.33	8.21 ± 4.62	7.88 ± 3.99
DLP (mGy·cm)	412 ± 256	431 ± 280	396 ± 227
Portal-venous phase			
kVp	100 / Sn150	100 / Sn150	100 / Sn150
Ref. mAs	171 ± 19 / 88.7 ± 16.1	170 ± 20 / 88.2 ± 13.6	172 ± 18 / 89.2 ± 18.1
mAs	234 ± 109 / 112 ± 59	234 ± 113 / 116 ± 74	234 ± 104 /109 ± 40
CTDI_vol_ (mGy)	13.4 ± 5.9	13.4 ± 6.0	13.3 ± 5.7
DLP (mGy·cm)	684 ± 376	705 ± 383	664 ± 365

Values are expressed as means ± standard deviation

*CTDI*_*vol*_ CT dose index, *DLP* dose-length product, *Sn* additional tin filtration

## Discussion

This study compared a conventional triphasic CTA protocol with a DECT protocol replacing true non-contrast images with VNC and corresponding iodine images. We demonstrated non-inferiority of the DECT protocol for sensitivity, specificity, and diagnostic accuracy and an improvement in diagnostic confidence.

Our results are in line with the only previous clinical study on DECT for GI bleeding [6, 7]. We were able to extend the results to show non-inferiority. Furthermore, we performed additional subgroup analyses. Both the training level of the readers and the location of the bleeding revealed non-inferior diagnostic accuracy, with a non-significant increase in accuracy with the DECT protocol. This was more pronounced for resident readers, possibly due to their overall lower diagnostic accuracy. While this was not investigated by Sun et al [6, 7], this is in accordance with results for DECT in patients with bowel ischemia, where less experienced residents benefit more from additional DECT reconstructions [11].

**Fig. 5:**
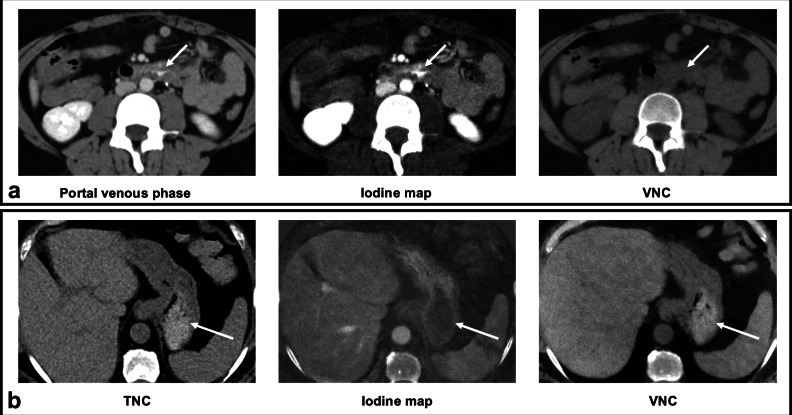
Diagnostic value of DECT-derived images compared to conventional images: **a** CT images of a patient with active arterial bleeding in the duodenum. At the same axial level, the contrast extravasation (white arrow) is more conspicuous in the iodine map than in the portal‑venous image, illustrating a possible reason for the higher sensitivity of the DECT protocol. The virtual non-contrast (VNC) image does not show any hyperdensity, confirming the contrast extravasation. **b** CT images of a different patient showing hyperdense material within the stomach (white arrow). The hyperdense intragastric material visible on the true non-contrast phase (TNC) is reproduced equally well on VNC, highlighting the interchangeability of VNC and TNC when correlating hyperattenuating findings from arterial or portal‑venous phases. At the same time, the iodine map does not show any hyperdensity

A possible mechanism behind the increase in diagnostic performance may be higher sensitivity due to the additional iodine map, where iodine intraluminal extravasations are easier to spot (Fig. 5). The higher diagnostic confidence may be due to the exact spatial correlation of the VNC and portal-venous phase image, unlike the conventional dataset, where the true non-contrast images show a temporal and spatial delay to the other contrast phases. Although this factor was not specifically assessed in the present study, it represents a possible explanation for the observed differences and could be tested in future research.

Reading time of the DECT dataset was also non-inferior to the conventional CT dataset, despite more images to review. This may possibly be due to a quicker decision because of higher confidence and the increased sensitivity due to the iodine maps as mentioned above.

While VNC can be viewed alone, ideally, material decomposition images should always be viewed as pairs to avoid false interpretation and allow for the recognition of artifacts [12].

We generated VNC and iodine maps from the portal-venous phase. Although an earlier consensus favored arterial-phase VNC, it was based on limited experience [13]. The portal-venous phase contains more pooled iodine, improving visualization of extravasation, and permits low-kVp arterial imaging, thereby reducing total dose. A recent photon-counting CT study likewise recommends portal-venous acquisitions for VNC generation [14].

Omitting the true non-contrast phase lowered radiation dose by approximately 20%, matching prior reports of 19–50% dose reductions when VNC replaces true non-contrast scans in abdominal triphasic DECT [7, 15, 16].

There are several limitations to our study. First, this was a retrospective, single-center study. For future studies, a multi-center study design would be desirable to achieve a more diverse patient and imaging collective. Unlike previous studies, our study added a non-inferiority design instead of only comparing conventional and DECT. Second, the case-control design prevents reliable estimation of positive and negative predictive values. Third, the gold standard for bleeding was not based on endoscopy. For two reasons, we chose to determine the reference standard based on the available imaging data and not based on endoscopy results. The first reason was to avoid verification bias since endoscopy was not performed in all cases [17]. The second reason was that the bleeding status can change between imaging and subsequent endoscopy. All scans were obtained on a dual-source DECT platform, so results may not translate directly to other DECT technologies (rapid kVp-switching and dual-layer spectral detector DECT), although their superior temporal alignment may yield comparable or better performance [18]. Fourth, readers may have identified bleeding in an incorrect location compared with the reference standard, resulting in a false-positive detection. The rate of such errors is expected to be lower than the observed rate of false-positive cases (bleeding detected by readers while no bleeding was present according to the reference standard). Given the low and comparable incidence of false-positive cases observed with both protocols, the likelihood of such occurrences should be minor and is unlikely to introduce bias.

Future studies on photon-counting detector CT would be desirable. For the reasons mentioned above, and additionally due to the improved spectral separation, better diagnostic performance for the detection of GI bleedings is expected.

In conclusion, when available, dual-source DECT should be used to replace true non-contrast images in triphasic CT protocols for suspected upper or lower GI bleeding. It increases diagnostic confidence while maintaining diagnostic accuracy without increasing reading time. Additionally, patients may benefit from lower radiation doses.

## Abbreviations

CTA: CT angiography
DLP: Dose-length product
DECT: Dual-energy CT
GI: Gastrointestinal
VNC: Virtual non-contrast

## References

[1] Expert Panels on Vascular Imaging and Gastrointestinal Imaging, Singh-Bhinder N, Kim DH et al (2017) ACR Appropriateness Criteria® nonvariceal upper gastrointestinal bleeding. J Am Coll Radiol 14:S177–S188. 10.1016/j.jacr.2017.02.038 28473074 10.1016/j.jacr.2017.02.038

[2] Expert Panel on Interventional Radiology, Karuppasamy K, Kapoor BS et al (2021) ACR Appropriateness Criteria® radiologic management of lower gastrointestinal tract bleeding: 2021 update. J Am Coll Radiol 18:S139–S152. 10.1016/j.jacr.2021.02.018 33958109 10.1016/j.jacr.2021.02.018

[3] Dane B, Gupta A, Wells ML et al (2023) Dual-energy CT evaluation of gastrointestinal bleeding. Radiographics 43:e220192. 10.1148/rg.220192 37167088 10.1148/rg.220192

[4] Nehra AK, Dane B, Yeh BM, Fletcher JG, Leng S, Mileto A (2023) Dual-energy, spectral and photon counting computed tomography for evaluation of the gastrointestinal tract. Radiol Clin North Am 61:1031–1049. 10.1016/j.rcl.2023.06.002 37758355 10.1016/j.rcl.2023.06.002

[5] May C, Sodickson A (2023) Leveraging dual-energy computed tomography to improve emergency radiology practice. Radiol Clin North Am 61:1085–1096. 10.1016/j.rcl.2023.06.003 37758358 10.1016/j.rcl.2023.06.003

[6] Sun H, Xue HD, Wang YN et al (2013) Dual-source dual-energy computed tomography angiography for active gastrointestinal bleeding: a preliminary study. Clin Radiol 68:139–147. 10.1016/j.crad.2012.06.106 22999524 10.1016/j.crad.2012.06.106

[7] Sun H, Hou XY, Xue HD et al (2015) Dual-source dual-energy CT angiography with virtual non-enhanced images and iodine map for active gastrointestinal bleeding: image quality, radiation dose and diagnostic performance. Eur J Radiol 84:884–891. 10.1016/j.ejrad.2015.01.013 25650332 10.1016/j.ejrad.2015.01.013

[8] Guglielmo FF, Wells ML, Bruining DH et al (2021) Gastrointestinal bleeding at CT angiography and CT enterography: imaging atlas and glossary of terms. Radiographics 41:1632–1656. 10.1148/rg.2021210043 34597220 10.1148/rg.2021210043

[9] Landis JR, Koch GG (1977) The measurement of observer agreement for categorical data. Biometrics 33:159–174 843571

[10] Committee for Proprietary Medicinal Products (2001) Points to consider on switching between superiority and non-inferiority. Br J Clin Pharm 52:223–228. 10.1046/j.0306-5251.2001.01397-3.x 10.1046/j.0306-5251.2001.01397-3.x PMC2014556 11560553

[11] Oberparleiter M, Vosshenrich J, Breit HC et al (2025) Dual-energy CT of acute bowel ischemia-influence on diagnostic accuracy and reader confidence. Eur Radiol 35:1405–1414. 10.1007/s00330-024-11217-1 39592488 10.1007/s00330-024-11217-1 PMC11836098

[12] Yeh BM, Obmann MM, Westphalen AC et al (2018) Dual energy computed tomography scans of the bowel: benefits, pitfalls, and future directions. Radiol Clin North Am 56:805–819. 10.1016/j.rcl.2018.05.002 30119775 10.1016/j.rcl.2018.05.002

[13] Patel BN, Alexander L, Allen B et al (2017) Dual-energy CT workflow: multi-institutional consensus on standardization of abdominopelvic MDCT protocols. Abdom Radiol (NY) 42:676–687. 10.1007/s00261-016-0966-6 10.1007/s00261-016-0966-6 27888303

[14] Risch F, Bette S, Sinzinger A et al (2023) Multiphase photon counting detector CT data sets—which combination of contrast phase and virtual non-contrast algorithm is best suited to replace true non-contrast series in the assessment of active bleeding? Eur J Radiol 168:111125. 10.1016/j.ejrad.2023.111125 37804649 10.1016/j.ejrad.2023.111125

[15] Trabzonlu TA, Mozaffary A, Kim D, Yaghmai V (2020) Dual-energy CT evaluation of gastrointestinal bleeding. Abdom Radiol (NY) 45:1–14. 10.1007/s00261-019-02226-6 10.1007/s00261-019-02226-6 31728614

[16] Baliyan V, Shaqdan K, Hedgire S, Ghoshhajra B (2019) Vascular computed tomography angiography technique and indications. Cardiovasc Diagn Ther 9:S14–S27. 10.21037/cdt.2019.07.04 31559151 10.21037/cdt.2019.07.04 PMC6732113

[17] Gennaro G (2018) The “perfect” reader study. Eur J Radiol 103:139–146. 10.1016/j.ejrad.2018.03.014 29653758 10.1016/j.ejrad.2018.03.014

[18] Obmann MM, Sun Y, An C, Ohliger MA, Wang ZJ, Yeh BM (2022) Bowel peristalsis artifact on dual-energy CT: in vitro study on the influence of different dual-energy CT platforms and enteric contrast agents. AJR Am J Roentgenol 218:290–299. 10.2214/AJR.21.26345 34406059 10.2214/AJR.21.26345

